# Silver Nanoparticles Potentiates Cytotoxicity and Apoptotic Potential of Camptothecin in Human Cervical Cancer Cells

**DOI:** 10.1155/2018/6121328

**Published:** 2018-12-12

**Authors:** Yu-Guo Yuan, Shimin Zhang, Ji-Yoon Hwang, Il-Keun Kong

**Affiliations:** ^1^College of Veterinary Medicine/Joint International Research Laboratory of Agriculture and Agri-Product Safety, the Ministry of Education of China, Yangzhou University, Yangzhou, Jiangsu 225009, China; ^2^Jiangsu Co-innovation Center for Prevention and Control of Important Animal Infectious Diseases and Zoonoses/Jiangsu Key Laboratory of Zoonosis, Yangzhou, Jiangsu 225009, China; ^3^Division of Applied Life Science (BK21 Plus), Institute of Agriculture and Life Science, Gyeongsang National University, Jinju 52828, Gyeongnam Province, Republic of Korea

## Abstract

Silver nanoparticles (AgNPs) are widely used metal nanoparticles in health care industries, particularly due to its unique physical, chemical, optical, and biological properties. It is used as an antibacterial, antiviral, antifungal, and anticancer agent. Camptothecin (CPT) and its derivatives function as inhibitors of topoisomerase and as potent anticancer agents against a variety of cancers. Nevertheless, the combined actions of CPT and AgNPs in apoptosis in human cervical cancer cells (HeLa) have not been elucidated. Hence, we investigated the synergistic combinatorial effect of CPT and AgNPs in human cervical cancer cells. We synthesized AgNPs using sinigrin as a reducing and stabilizing agent. The synthesized AgNPs were characterized using various analytical techniques. The anticancer effects of a combined treatment with CPT and AgNPs were evaluated using a series of cellular and biochemical assays. The expression of pro- and antiapoptotic genes was measured using real-time reverse transcription polymerase chain reaction. The findings from this study revealed that the combination of CPT and AgNPs treatment significantly inhibited cell viability and proliferation of HeLa cells. Moreover, the combination effect significantly increases the levels of oxidative stress markers and decreases antioxidative stress markers compared to single treatment. Further, the combined treatment upregulate various proapoptotic gene expression and downregulate antiapoptotic gene expression. Interestingly, the combined treatment modulates various cellular signaling molecules involved in cell survival, cytotoxicity, and apoptosis. Overall, these results suggest that CPT and AgNPs cause cell death by inducing the mitochondrial membrane permeability change and activation of caspase 9, 6, and 3. The synergistic cytotoxicity and apoptosis effect seems to be associated with increased ROS formation and depletion of antioxidant. Certainly, a combination of CPT and AgNPs could provide a beneficial effect in the treatment of cervical cancer compared with monotherapy.

## 1. Introduction

Cancer is a leading cause of death worldwide among women in both high-income countries and middle-income countries [1]. Females are easily afflicted by cancer, which is the second leading cause of death worldwide, accounting for 14% of all deaths. According to the World Health Organization (WHO) International Agency for Research on Cancer (IARC), there were 6.7 million new cancer cases and 3.5 million deaths among females worldwide in 2012 [2]. The numbers of cases are expected to increase to 9.9 million cases and 5.5 million deaths among females annually by 2030 as a result of the growth and aging of the population [2]. Cervical cancer exhibited with an estimated 527,600 cases and 265,700 deaths among women worldwide in 2012. In developed countries like the USA, 12,990 women will be newly diagnosed with cervical cancer and 4120 will die from the disease in 2016 [1]. In developed countries, cervical cancer is the fourth leading cause of cancer, whereas in developing countries, it is the second most commonly diagnosed cancer after breast cancer and the third leading cause of cancer death after breast and lung cancers [2]. In fact, almost 90% of cervical deaths in the world occur in developing countries, with India alone accounting for 25% of the total cases. To prevent the occurrence of cervical cancer, several modalities have been established such as screening, vaccination, electrosurgical excision procedure, cryotherapy, surgery, radiation, and chemotherapy or combination of chemo and radiation or combination of chemo and nanoparticles.

Combination therapy is using two or more therapeutic agents to increase the efficacy of drug using low concentration and to reduce drug resistance in cancer cells by chemosensitization by additive or synergistic effects. The foundation of combination therapy is specifically targeting providing treatments for cancer-inducing or cell-sustaining pathways [3, 4]. Monotherapy nonselectively target proliferating cells which leads to the destruction of both healthy and cancerous cells. Generally, chemotherapy exhibited undesired side effects and risks and can also strongly reduce their immune system by affecting bone marrow cells and increasing susceptibility to host diseases [5, 6]. Although combination therapy seems to be toxic, it can be overcome by using two different chemotherapeutic agents by using low concentrations and working on two different mechanisms to control the proliferation of cells. Particularly, the combination of anticancer drug and biocompatible nanoparticles is able to reduce the undesired side effects. Furthermore, combination therapy may be able to prevent the toxic effects on normal cells while simultaneously producing cytotoxic effects on cancer cells and combat expected acquired resistance or minimize the possibility for development of drug resistance. Recently, combinations of two different chemotherapeutic agents are currently favorable to debulking the tumor mass; however, these methods often produce side effects and are insufficient to cure cancer patients and relapse commonly follows due to clinically undetectable micrometastases [7].

Chemotherapy seems to be an effective treatment to kill cancer cells by using various anticancer drugs such as Pt-based drugs, nitrogen mustards, and drugs like temozolomide that are based on DNA alkylation, which causes severe side effects in many types of cancer [8]. Particularly, platinum-based drugs such as cisplatin exert anticancer effects by multiple mechanisms; surprisingly, cisplatin treatment often leads to the development of chemoresistance, thereby causing therapeutic failure. Carboplatin is considered to be less toxic and nonhematologic toxicity than the parent molecule. A combination of carboplatin and paclitaxel was used to treat solid malignancies, resulting in less platelet toxicity than treatment with carboplatin alone [9]. Camptothecin (CPT) is an anticancer agent and has unique mechanism of action, which is the reversible inhibition of DNA topoisomerase I. The combination of CPTs and topoisomerase II inhibitors has synergistic activity in a variety of cancer cell lines by the mechanism of chromosomal aberrations including increasing sister chromatid exchanges, gene deletions, and gene rearrangements [10]. CPTs can enhance radiation-induced cytotoxicity and also show substantial time- and dose-dependent radio-sensitization [11, 12]. Ciesielski and Fenstermaker demonstrated that subcytotoxic doses of CPT not only potentiated the action of VP-16 in glioma cell line (U87) but also increased significant synergistic effect with all topo II inhibitors such as doxorubicin, ellipticine, and m-AMSA [13].

Combinations of chemotherapeutic agents and nanoparticle have shown much interest in cancer treatment. Combined therapy of anticancer drugs and nanoparticles promotes synergism and suppresses drug resistance through distinct mechanisms of action at a low dose. Nanoparticle-mediated combination therapy increases the efficacy of anticancer, reduces undesired side effects and improves pharmacokinetics [14]. Nanoparticles mediated combination therapy to induce cytotoxicity comprising various kind of approaches such as nanoparticle as delivery agent for siRNA, codelivery of chemical and siRNA by a single nanoparticle, use of multiple nanoparticles for chemical and siRNA therapeutics, sequential administration of single anticancer agent and selective nanoparticles and sequential administrations of multiple anticancer drugs with multiple nanoparticles. Furthermore, nanoparticles can be used as direct cytotoxic agents with an anticancer drug like salinomycin [15] and also a combination of two or more nanoparticles with different pharmacological actions may synergistically decrease cell viability. For instance, the sequential administration of erlotinib and DOX increase the apoptotic signaling pathway and increase antineoplastic effects on the triple-negative BT-20 cells [16]. Zhang et al. [17] demonstrated the codelivery of the cytotoxic DOX with the antivasculature agent combretastatin A4 (CA4) induce the destruction of vascular walls and facilitate the extraversion of post released DOX, thus increasing the therapeutic DOX efficacy. Recently, Zhang et al. [18] demonstrated that the combination of trichostatin A and palladium nanoparticles exhibited dose-dependent synergistic effect in human cervical cancer cells. The combination of TSA and PdNPs had a more pronounced effect on cytotoxicity by strong synergistic interaction between TSA and PdNPs in cervical cancer cells. Similarly, graphene oxide AgNPs nanocomposite showed pronounced synergistic inhibitory effect with trichostatin A in human ovarian cancer cells [19].

Due to excellent potential properties of physical, chemical, and biological, AgNPs are known to exhibit antibacterial, antiviral, anti-inflammatory, anticancer, and antiangiogenic activity [20]. AgNPs are able to modulate the Pgp activity and able to enhance chemotherapeutic efficacy in multidrug resistant cancer cells, and also it seems to be potential combinational partners with the anticancer drug [21, 22]. Furthermore, the mechanism of toxicity of apoptosis induced by AgNPs is well established in a variety of cell lines such as human lung cancer cells, ovarian, and breast cancer. Therefore, AgNPs are the best, suitable, and alternative nanoparticles working with any anticancer drug. CPT is a broad spectrum of anticancer agent, which is capable of inducing apoptosis through blocking the advancement of replication forks [23].

Here, we hypothesize that the multifunctional nature of AgNPs could contribute pronounce anticancer activity of CPT; therefore, we designed the following objectives. Firstly, we synthesized AgNPs using novel biomolecule called sinigrin. Secondly, we evaluated the cytotoxic potential of coadministration of anticancer agent CPT and AgNPs. Thirdly, evaluation of the underlying mechanism of cytotoxicity caused by CPT and AgNPs was used to measure the expression of oxidative and antioxidative stress markers. Finally, we analyzed the expression of apoptotic and antiapoptotic genes and genes involved in the important signaling pathway in human cervical cancer cells. This novel combinational formulation ascertains to be a powerful tool to overcome chemoresistance by enhancing cytotoxicity and apoptosis.

## 2. Materials and Methods

### 2.1. Reagents

Penicillin-streptomycin, trypsin-EDTA, Dulbecco's modified Eagle's medium (DMEM), RPMI 1640 medium, and 1% antibiotic-antimycotic were obtained from Life Technologies/Gibco (Grand Island, NY, USA). Sinigrin, carboplatin, CPT, fetal bovine serum (FBS), and the *in vitro* toxicology assay kit were purchased from Sigma-Aldrich (St. Louis, MO, USA). Silver nitrate and all other chemicals were purchased from Sigma-Aldrich unless otherwise stated.

### 2.2. Synthesis of AgNPs

The synthesis of AgNPs was performed using sinigrin as described previously [23]. AgNPs were prepared by two different approaches, the first approach was by adding 1 mL of 1 mg sinigrin to 10 mL 1 mM aqueous AgNO_3_; the mixture was incubated for 6 h at 40°C and pH 8.0. The second type of approach was by adding 1 mL of 1 mg Sinigrin to 10 mL 5 mM aqueous AgNO_3_; the mixture was incubated for 12 h at 60°C and pH 8.0. The bioreduction of the silver ions was monitored spectrophotometrically at 420 nm. Further characterizations of the synthesized AgNPs with TEM were performed as described previously [24].

### 2.3. Cell Culture, Cell Viability, and Measurement of LDH and ROS

Human cervical cancer cells (HeLa) (ATCC® CCL2™), obtained from ATCC, were cultured in RPMI-1640 medium supplemented with 10% fetal bovine serum and 1% penicillin-streptomycin (Gibco BRL, Grand Island, NY) and were maintained in a humidified incubator at 5% CO_**2**_ and 37°C. Cells were routinely grown in 100 mm plastic tissue culture dishes (Nunc, Roskilde, Denmark) and harvested with a solution of trypsin-EDTA, while in a logarithmic phase of growth. The CCK-8 and LDH assays were performed as described previously [25, 26]. Cell membrane integrity of the human cervical cancer cells was evaluated by determining the activity of lactate dehydrogenase (LDH) leaking out of the cells, as per the manufacturer's instructions and also as described previously [25]. ROS were estimated according to a method described previously [27].

### 2.4. Cell Proliferation Assay

Cell proliferation was evaluated according to the manufacturer's instructions using BrdU cell proliferation assay kit. HeLa cells were plated in 6-well plates (1 × 10^5^ cells per well) and incubated for 24 h. CPT (1 *μ*M) or AgNPs (1 *μ*M) or a combination of CPT (1 *μ*M) and AgNPs (1 *μ*M) was then added to the cells for 24 h. Cells cultured in the medium without CPT or AgNPs were used as controls.

### 2.5. Trypan Blue Exclusion Assay

A trypan blue exclusion assay was performed according to the method described earlier [28]. HeLa cells were plated in 96-well plates. The cells were treated with CPT (1 *μ*M) or AgNPs (1 *μ*M) or a combination of CPT (1 *μ*M) and AgNPs (1 *μ*M) for 24 h. After treatment, cells attached to the 96-well plate were washed using phosphate-buffered saline (PBS, pH 7.4) once, trypsinized for 2 min in a 37°C incubator, and then neutralized with fetal bovine serum- (FBS-) supplemented growth media. The cells were stained using 4% trypan blue to determine live cell numbers. The cell count was performed manually with a hemocytometer.

### 2.6. Cell Morphology

Human cervical cancer cells were plated in 6-well plates (2 × 10^5^ cells per well) and incubated with 1 *μ*M CPT or 1 *μ*M AgNPs for 24 h. Cells cultured in medium without the addition of CPT or AgNPs were used as the control. The cell morphology was analyzed using an optical microscope at 24 h posttreatment. The morphology of the cells was examined with an OLYMPUS IX71 microscope (Japan) using the appropriate filter sets.

### 2.7. Determination of Reactive Oxygen Species (ROS)

ROS were estimated according to a method described previously. Intracellular ROS were measured based on the intracellular peroxide-dependent oxidation of 2′,7′-dichlorodihydrofluorescein diacetate (DCFH-DA, Molecular Probes, NY, US) to form the fluorescent compound 2′,7′-dichlorofluorescein (DCF), as previously described. The cells were seeded onto 24-well plates at a density of 5 × 10^4^ cells per well and cultured for 24 h. After washing twice with PBS, fresh media containing CPT (1 *μ*M) or AgNPs (1 *μ*M) or a combination of CPT (1 *μ*M) and AgNPs (1 *μ*M) was then added to the cells for 24 h. The cells were then supplemented with 20 *μ*M DCFH-DA, and the incubation continued for 30 min at 37°C. The cells were rinsed with PBS, and 2 mL of PBS was added to each well. The fluorescence intensity was determined using a spectrofluorometer (Gemini EM, Molecular Devices, Sunnyvale, CA, USA) with excitation at 485 nm and emission at 530 nm.

### 2.8. Membrane Integrity

The membrane integrity of HeLa cells was evaluated according to the manufacturer's instructions (LDH Cytotoxicity Detection Kit; Takara, Tokyo, Japan). Briefly, the cells were treated with CPT (1 *μ*M) or AgNPs (1 *μ*M) or a combination of CPT (1 *μ*M) and AgNPs (1 *μ*M) for 24 h and 100 *μ*L per well of cell-free supernatant was transferred in triplicate into 96-well plates. Then, 100 *μ*L of the LDH reaction mixture was added to each well. After 3 h incubation under standard conditions, the optical density of the color generated was determined at a wavelength of 490 nm using a microplate reader.

### 2.9. Assessment of Dead-Cell Protease Activity

A dead-cell protease activity assay was performed according to the method described earlier [29]. The cytotoxicity of CPT (1 *μ*M) or AgNPs (1 *μ*M) or a combination of CPT (1 *μ*M) and AgNPs (1 *μ*M) for 24 h was determined by the association of intracellular protease with a luminogenic peptide substrate (alanyl-alanylphenylalanyl-aminoluciferin). The degree of protease reaction can measure dead-cell protease activity. As a control, we treated cells with 1% Triton X-100 to exclude the background value of the medium color. Luminogenic peptide substrate (5 *μ*L) was added to each well, and luminescence was measured to determine the number of dead cells. The peptide substrate was incubated for 15 min at 37°C. The luminescence was measured with a luminescence counter (PerkinElmer, Waltham, MA, USA).

### 2.10. Measurement of Oxidative and Antioxidative Stress Markers

The expression level of oxidative stress markers and antioxidative stress markers was measured as described previously [29, 30]. MDA was measured according to the method described earlier [28]. The HeLa cells were seeded into six-well microplates at 2.0 × 10^5^ cells per well. The cells were treated with CPT (1 *μ*M) or AgNPs (1 *μ*M) or a combination of CPT (1 *μ*M) and AgNPs (1 *μ*M) for 24 h. After incubation, the cells were harvested and washed twice with an ice-cold PBS solution. The cells were collected and disrupted by ultrasonication for 5 min on ice. The cell extract (100 *μ*L) was used to detect MDA according to the procedure recommended by the manufacturer of the MDA assay kit. The concentration of MDA was measured on a microplate reader at a wavelength of 530 nm. The protein concentration was determined using the Bio-Rad protein assay kit (Bio-Rad, Hercules, CA, USA). Carbonyl content in oxidatively modified proteins was determined by the method of Levine et al. [31] and also as described by the manufacturer's instructions from Sigma-Aldrich. Antioxidative stress markers, such as glutathione (GSH), superoxide dismutase (SOD), catalase (CAT), and glutathione peroxidase (GPx) were assayed with reagents from various kits, according to each manufacturer's instructions (Sigma). Briefly, the cells were cultured in 75 cm^2^ culture flasks and exposed to CPT or AgNPs or a combination of both for 24 h. The cells were then harvested in chilled PBS, by scraping and washing twice with 1× PBS at 4°C for 6 min at 1500 rpm. The cell pellet was sonicated at 15 W for 10 s (3 cycles) to obtain the cell lysate. The resulting supernatant was stored at −70°C until analyzed.

### 2.11. Measurement of Mitochondrial Dysfunction

MMP was measured, as described previously using a cationic fluorescent indicator JC-1 (Molecular Probes, Eugene, OR, USA) [26]. Briefly, the cells were cultured in 75 cm^2^ culture flasks and were exposed to CPT (1 *μ*M) or AgNPs (1 *μ*M) or a combination of CPT (1 *μ*M) and AgNPs (1 *μ*M) for 24 h, and then MMP was measured as described previously using the cationic fluorescent indicator JC-1 (Molecular Probes, Eugene, OR, USA) [26]. JC-1 is a lipophilic cation, which, in a reaction driven by Δ*ψ*m in normal polarized mitochondria, assembles into a red fluorescence-emitting dimer, forming JC-1-AgNPs aggregates. Cells were incubated with 10 *μ*M JC-1 at 37°C for 15 min, washed with PBS, resuspended in PBS, and then the fluorescence intensity was measured. MMP was expressed as the ratio of the fluorescence intensity of the JC-1 aggregates to that of the monomers.

### 2.12. Quantitative RT-PCR Analysis

According to the manufacturer's instructions, total RNAs was extracted from treated and untreated cells using Dynabeads mRNA Direct Kit (Thermo Fisher, USA). Real-time qRT-PCR was conducted using a Vill7 (Applied Biosystems, OR, USA) and SYBR Green as the double-stranded DNA-specific fluorescent dye (Applied Biosystems, OR, USA). Target gene expression levels were normalized to *GAPDH* gene expression, which was unaffected by CPT (1 *μ*M) or AgNPs (1 *μ*M) or a combination of CPT (1 *μ*M) and AgNPs (1 *μ*M). The real-time qRT-PCR primer sets are shown in Tables 1 and 2. Real-time qRT-PCR was performed independently in triplicate, for each of the different samples, and the data are presented as the mean values of the gene expression levels measured in the treated samples versus the controls.

### 2.13. Statistical Analysis

All assays were conducted in triplicate, and each experiment was repeated at least 3 times. The results represent the mean of at least 3 independent experiments (mean ± SD). The Student's *t*-test or one-way analysis of variance (ANOVA), followed by Tukey's test for multiple comparisons, were calculated using the GraphPad Prism software (GraphPad Software, San Diego, CA, USA). The differences were considered significant at *p* < 0.05.

## 3. Results and Discussion

### 3.1. Synthesis and Characterization of AgNPs Using Sinigrin

**Figure 1:**
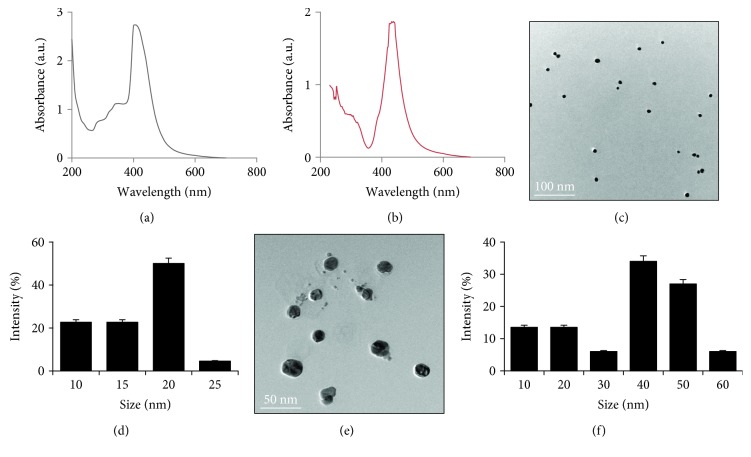
Synthesis and characterization of AgNPs using sinigrin. (a) The absorption spectrum of 20 nm size AgNPs. (b) The absorption spectrum of 40 nm size AgNPs. (c) TEM images of 20 nm size of AgNPs. (d) Several fields of TEM images were used to measure the particle size of AgNPs; histogram shows size distributions based on TEM images of AgNPs ranging from 10 to 25 nm. (e) TEM images of 40 nm size of AgNPs. (f) Several fields of TEM images were used to measure the particle size of AgNPs; histogram shows size distributions based on TEM images of AgNPs ranging from 10 to 60 nm.

Size and shape of the nanoparticles are important factors for biological activity. Recently, synthesis of AgNPs using a variety of green reducing agents are fashion and prominent. Green synthesis of AgNPs was performed by using a cellular extract of bacteria, fungi, plants and purified proteins, polyphenols, alkaloids, flavonoids, and so on [15, 28, 29, 32]. In this study, we explored the possibility of unexplored glucosinolate compound such as sinigrin for the synthesis of AgNPs. For the synthesis of AgNPs, two different approaches were selected. The first approach was by adding 1 mL of 1 mg sinigrin to 10 mL 1 mM aqueous AgNO_3_; the mixture was incubated for 6 h at 40°C and pH 8.0. The second type of approach was by adding 1 mL of 1 mg sinigrin to 10 mL 5 mM aqueous AgNO_3_; the mixture was incubated for 12 h at 60°C and pH 8.0. The optical properties of spherical AgNPs are highly dependent on the nanoparticle diameter. Smaller spherical AgNPs primarily absorbed light and had peaks near 400 nm, while larger nanoparticles exhibited increased scattering and had peaks that broadened and shifted towards longer wavelengths at 425 nm (Figures 1(a) and 1(b)). The bioreduction of the silver ions was monitored spectrophotometrically at 420 nm. Both approaches produced two different sizes of AgNPs with an average size of 40 nm and 20 nm, respectively (Figures 1(c)–1(f)). The physico-chemical properties of AgNPs are important factor that increased bioavailability and toxicity of engineered NPs compared to corresponding micro size compounds [33]. Although a variety of nanoparticles have been synthesized using a variety of reducing agents, the studies on toxicological implications of glucosinolate-mediated synthesis of AgNPs with anticancer drug in human cervical cancer cells are not known, and it is very interesting to find out the mechanism of action and its applications in nanomedicine. Therefore, further studies were performed using these two different sizes of AgNPs derived from sinigrin in human cervical cancer cells.

### 3.2. Effect of Two Different Sizes of Sinigrin-Mediated Synthesis of AgNPs on Human Cervical Cancer Cells

**Figure 2:**
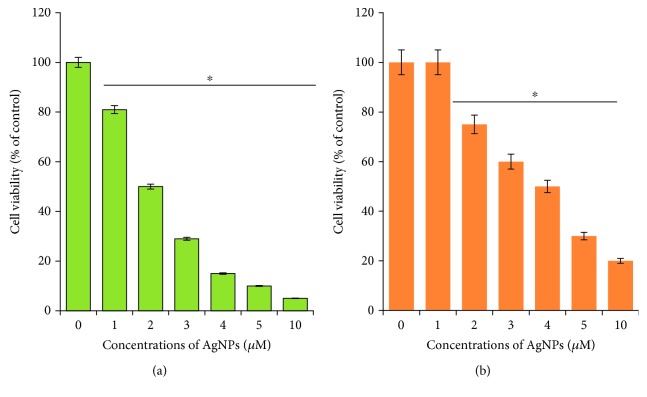
Dose-dependent effect of AgNPs 20 and 40 nm in HeLa cells. (a) HeLa cells were incubated with various concentrations of 20 nm AgNPs (1–10 *μ*M) for 24 h, and cell viability was measured using CCK-8. (b) HeLa cells were incubated with various concentrations of 40 nm AgNPs (1–10 *μ*M) for 24 h, and cell viability was measured using CCK-8. The results are expressed as the mean ± standard deviation of three separate experiments. Differences between the treated and control groups were measured using Student's *t*-test. Statistically significant differences between the treated and control group are indicated by (^∗^*p* < 0.05).

To determine the efficacy of two different sizes of AgNPs in cervical cancer cells, CCK-8 assays were performed according to the method described earlier [23]. Cells were plated at a density of 1 × 10^5^ cells/well in a 96-well culture plate and cultured overnight to allow adherence and recovery. After this period, nonattached cells were aspirated and different concentrations (1–10 *μ*M) of both 20 and 40 nm sizes of AgNPs were added and then incubated for 24 h. As expected, the cell viability loss was observed in both tested AgNPs, which were exhibited in size and dose-dependent manner (Figures 2(a) and 2(b)). The significant toxicity was observed in the smaller size of AgNPs, and less effect was observed with 40 nm than 20 nm at particular concentrations. AgNPs with an average size of 40 nm exhibited toxicity at higher concentrations, which could be due to slow release of silver ions by the larger size of AgNPs. Carlson et al. [34] evaluated size-dependent cellular interactions of three different sizes of AgNPs such as Ag-15 nm, Ag-30 nm, and Ag-55 nm in alveolar macrophages. The study suggests that smaller size such as 15 nm causes severe toxicity by oxidative stress. AgNPs are known to induce proinflammatory responses in primary rat brain microvessel endothelial cells (rBMEC). Among the three tested different sizes of 25, 40, and 80 nm AgNPs, AgNPs with an average size of 25 and 40 nm induced cytotoxic responses at lower concentrations [35]. Park et al. [36] demonstrated the size-dependent cytotoxicity, inflammation, genotoxicity, and developmental toxicity in L929 fibroblasts. The results showed that 20 nm AgNPs were more toxic than the larger nanoparticles and silver ions. The toxicity of AgNPs depends on the potency of silver cell type and size-dependent. The size-dependent cellular response of AgNPs (10, 20, 40, 60, and 100 nm) was performed in human LoVo cell line. The results indicate that toxicity is size- and dose-dependent manner. Particularly, smaller particles have easily penetrated the cells than larger size [37]. BEAS-2B cells were exposed to various sizes of AgNPs (10, 40, and 75 nm), small AgNPs (10 nm) are cytotoxic for human lung cells, and the toxicity was merely associated with the rate of intracellular Ag release, a “Trojan horse” effect [38]. Gurunathan et al. demonstrated that the toxicity of AgNPs is not only size-dependent but also the type of capping agent used for the synthesis of AgNPs [39]. For instance, fungal extract capped AgNPs showed more toxicity than bacterial extract capped AgNPs in human breast cancer cells. Collectively, 20 nm AgNPs showed a significant toxicity than 40 nm. In order to use subcytotoxic dose for a combination study with anticancer drug, we measured IC_25_ values for 20 nm and 40 nm. The IC_25_ values of 20 nm and 40 nm were found to be 1 *μ*M and 2 *μ*M, respectively. Further experiments were carried out using these concentrations to depict the potential effect of AgNPs with the anticancer drug in human cervical cancer cells. Therefore, for combination experiments with CPT, we selected only 20 nm sizes of AgNPs with an IC_25_ value of 1 *μ*M for further experiments.

### 3.3. Effect of Carboplatin and CPT on Cell Survival of HeLa Cells

**Figure 3:**
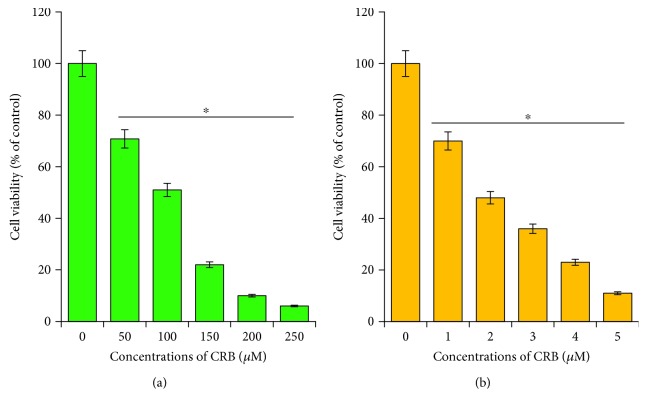
Dose-dependent effect of carboplatin and camptothecin in HeLa cells. (a) HeLa cells were incubated with various concentrations of carboplatin (50–250 *μ*M) for 24 h, and cell viability was measured using CCK-8. (b) HeLa cells were incubated with various concentrations of camptothecin (1–5 *μ*M) for 24 h, and cell viability was measured using CCK-8. The results are expressed as the mean ± standard deviation of three separate experiments. Differences between the treated and control groups were measured using Student's *t*-test. Statistically significant differences between the treated and control group are indicated by (^∗^*p* < 0.05).

Carboplatin is one of the main platinum-based drugs used as an anticancer agent, which is working on the principles of ability to generate lesions in DNA through the formation of adducts with platinum, thereby inhibiting replication and transcription and leading to cell death [40]. CPT and its analogs stabilize the DNA-topoisomerase I cleavable complex and cause accumulation of single-stranded breaks in DNA [41]. CPT and its analogs are known to induce cytotoxic effects by inducing caspase activation, cell surface Fas receptor activation, and ROS formation [42]. Carboplatin and CPT are used as anticancer drug in a variety of cancers including breast, ovarian, lung, and cervical cancers. Therefore, we evaluated the potential toxicity in HeLa cells. We selected a range of dose for both carboplatin and CPT. The cells were treated with carboplatin between 50 and 250 *μ*M and CPT between 1 and 5 *μ*M for 24 h. The results showed that both carboplatin and CPT displayed a dose-dependent effect in HeLa cells (Figures 3(a) and 3(b)). Interestingly, CPT exhibited a significant toxicity at lower concentrations compared to carboplatin. Elsayed et al. [43] reported that combination of CPT with other anticancer drugs such as carboplatin, paclitaxel, doxorubicin and mitomycin, or signaling inhibitors (farnesyltransferase inhibitor and ERK inhibitor) did not enhance the CPT-induced cell death and caspase-3 activation. Therefore, we selected the combination between CPT and smaller size of 20 nm AgNPs for all experiments.

### 3.4. Dose-Dependent Combination Effect of CPT and AgNPs on HeLa Cells

**Figure 4:**
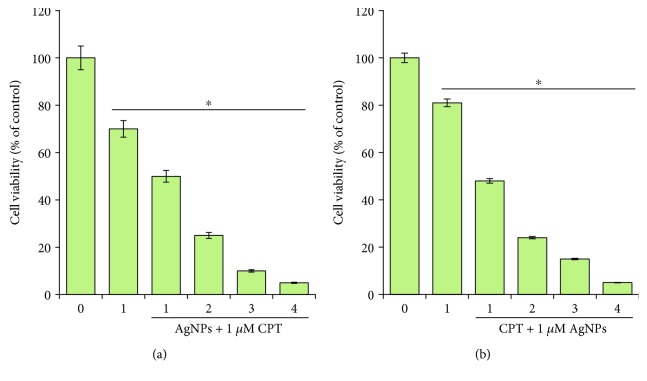
The effect of combined treatment with camptothecin and AgNPs on cell viability in HeLa cells. (a) HeLa cells were incubated with a combination of different concentrations of AgNPs (1–4 *μ*M) and a fixed concentration of CPT (1–4 *μ*M) for 24 h, and cell viability was measured using CCK-8. The results are expressed as the mean ± standard deviation of three separate experiments. (b) HeLa cells were incubated with a combination of different concentrations of CPT (1–4 *μ*M) and a fixed concentration of AgNPs (1–4 *μ*M) for 24 h, and cell viability was measured using CCK-8. The results are expressed as the mean ± standard deviation of three separate experiments. Differences between the treated and control groups were measured using Student's *t*-test. Statistically significant differences between the treated and control group are indicated by (^∗^*p* < 0.05).

The cytotoxic potential of the combination of AgNPs and CPT against cancer cells is still obscure. To further confirm the cooperative or additive effect of CPT and AgNPs, an experiment was performed to determine dose-dependent combination effect of CPT and AgNPs in HeLa cells which was measured by analysis of cell viability by CCK-8. When HeLa cells were treated with various concentrations of AgNPs with 1 *μ*M CPT, cell viability decreased in a concentration-dependent manner. The incidence of cell death after treatment with 1, 2, 3, and 4 *μ*M of AgNPs and 1 *μ*M CPT for 24 h was about 50%, 75%, 90%, and 95%, respectively (Figure 4(a)). Similarly, when HeLa cells were treated with various concentrations of CPT and 1 *μ*M AgNPs, cell viability also decreased in a concentration-dependent manner. The incidence of cell death after treatment with 1, 2, 3, and 4 *μ*M of CPT and 1 *μ*M AgNPs for 24 h was about 52%, 76%, 85%, and 95%, respectively (Figure 4(b)). In either case, the loss of cell viability trend was similar. Combined treatment of AgNPs with anticancer drugs CPT did augment CPT toxicity. CPT analogs have been shown to cause cell death in cancer cells by inducing the activation of apoptosis effector caspases [42, 44]. Combination of CPT- and VP-16-induced apoptosis in human leukemia HL60 and U937 cells [45]. Clinical trials were carried out using a combination of topotecan with other active agents, such as cisplatin or paclitaxel in patients with earlier stages of ovarian cancer [46–49]. Recently, AgNPs showed enhanced apoptotic and autophagy activity by combination with salinomycin [15], and the combination of gemcitabine and AgNPs increased apoptotic potential by increasing oxidative stress, loss of mitochondrial membrane potential, and increased the expression proapoptotic and downregulation of antiapoptotic genes in human ovarian cancer cells. Collectively, AgNPs is the suitable, biocompatible, and alternative combinatorial agent to increase anticancer activity.

### 3.5. Antiproliferative Effect of CPT and AgNPs on HeLa Cells

**Figure 5:**
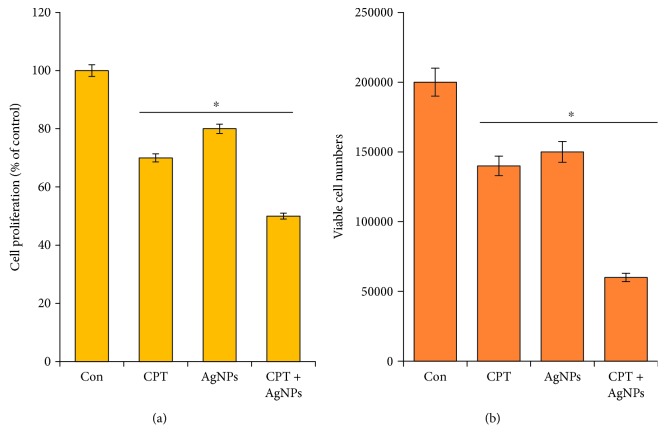
The effect of combined treatment with CPT and AgNPs on the proliferation of HeLa cells. (a) The effect on cell proliferation was observed by measuring the incorporation of BrdU after a 24 h incubation with CPT (1 *μ*M), AgNPs (1 *μ*M), or a combination of CPT (1 *μ*M) and AgNPs (1 *μ*M). (b) The effect on cell proliferation was observed using the trypan blue exclusion assay after 24 h incubation with CPT (1 *μ*M), AgNPs (1 *μ*M), or a combination of CPT (1 *μ*M) and AgNPs (1 *μ*M). The results are expressed as the mean ± standard deviation of three independent experiments. Differences between the treated and control groups were measured using Student's *t*-test. Statistically significant differences between the treated and control group are indicated by (^∗^*p* < 0.05).

We compared the ability of CPT or AgNPs alone or a combination of both CPT and AgNPs on the proliferation of HeLa cells using subtoxic concentration of CPT and AgNPs (1 *μ*M each) for 24 h by BrdU and trypan blue exclusion assay. As shown in Figures 5(a) and 5(b), HeLa cells were found to be the most sensitive to both CPT and AgNPs. The cell's proliferation decreased after treated with CPT or AgNPs; however, the effect was more pronounced in the presence of both CPT and AgNPs. The combination of CPT and AgNPs significantly reduced the proliferation efficiency of HeLa cells when compared with single treatment alone. The different effect of CPT and AgNPs was detectable in terms of maximal inhibition of cell proliferation and effective doses. Interestingly, both BrdU and trypan blue assays show a similar pattern of the antiproliferative effect of CPT and AgNPs. For instance, b-cyclodextrin-nanosponges (CN-CPT) significantly inhibited cell viability, clonogenic capacity, and cell-cycle progression of ATC cell lines compared to free CPT. When vascular endothelial cells are treated with CN-CPT, it inhibited adhesion, migration, secretion of proangiogenic factors (IL-8 and VEGF-a), and also inhibited phosphorylation of the Erk1/2 MAPK. CN-CPT significantly inhibited the growth, the metastatization, and the vascularization of orthotopic ATC xenografts in SCID/beige mice without apparent toxic effect in vivo [50]. Similarly, AgNPs inhibit proliferation, cell viability, migration, and phosphorylation of Akt in bovine retinal endothelial cells [51, 52]. Collectively, the combination of CPT and AgNPs effectively inhibited the proliferation of HeLa cells. It indicated that HeLa cells showed pronouncedly decreased cell viability after treated with CPT and AgNPs compared to CPT or AgNPs alone.

### 3.6. Combination of Camptothecin and AgNPs Alters Cell Morphology

**Figure 6:**
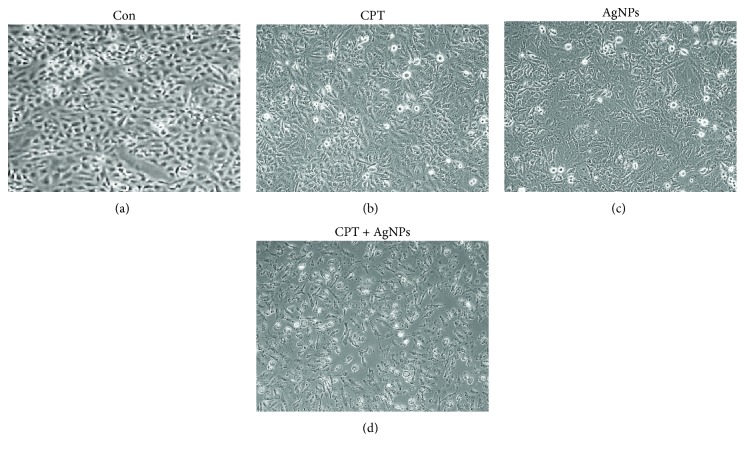
The effect of single treatment with CPT or AgNPs, or the combination of CPT and AgNPs, on cell morphology of HeLa cells. HeLa cells were incubated with CPT (1 *μ*M), AgNPs (1 *μ*M), or a combination of CPT (1 *μ*M) and AgNPs (1 *μ*M) for 24 h. Treated cells were imaged under a light microscope (200 *μ*m).

To determine whether CPT, AgNPs, or the combination of CPT and AgNPs could influence cell morphology, we evaluated HeLa cells treated with CPT (1 *μ*M), AgNPs (1 *μ*M), or both CPT and AgNPs (1 *μ*M plus 1 *μ*M). AgNPs are known to cause toxicity by morphological changes that appeared in a variety of cancer cells including human lung cancer and ovarian [25, 27, 53]. Human hepatoma cells displayed abnormal morphology, cellular shrinkage, detachment, decreased mitochondrial function, and significantly increased LDH after 24 h of exposure to AgNPs [54]. Treatment with either CPT or AgNPs alone caused marginal cell morphological changes, such as a round shape, whereas the combination of CPT and AgNPs caused severe morphological changes, such as rounder cells and lower cell density than in either single treatment group (Figure 6). Loss of normal cell morphology was observed in cells treated with CPT or AgNPs alone after 24 h of exposure. The detached cells became spherical in shape, forming clusters, and eventually detached from the surface. The control cells showed a high confluency of monolayer cells compared to either CPT- or AgNPs-treated cells, whereas CPT- and AgNPs-treated cells showed a reduction in cell volume and cell shrinkage and finally resulted in the generation of apoptotic bodies. Acevedo-Morantes et al. [55] observed a significant cell morphology loss after 10 h of treatment of MCF7 cells at concentrations higher than 0.5 mM of CPT:SLN complexes in a longer time exposure. Recently, the study reported that the combination of gemcitabine and AgNPs caused severe loss of cell morphology by decreasing the cell intensity and losing their adherence to the surface and displaying intensive blebbing, which was typical for apoptotic cell death [27]. The combination of CPT and AgNPs also caused severe cell shrinkage and efflux of cytoplasmic content. Cell shrinkage is a characteristic feature of apoptosis and is caused by disruption of the maintenance of the normal physiological concentrations of K^+^ and Na^+^ and intracellular ion homeostasis [56]. Taken together, CPT and AgNPs altered cellular morphology and appreciable cell morphology loss, which was in accordance with previously published reports and could provide evidence for their toxicity.

### 3.7. Combination of CPT and AgNPs Cause Severe Cytotoxicity in HeLa Cells

**Figure 7:**
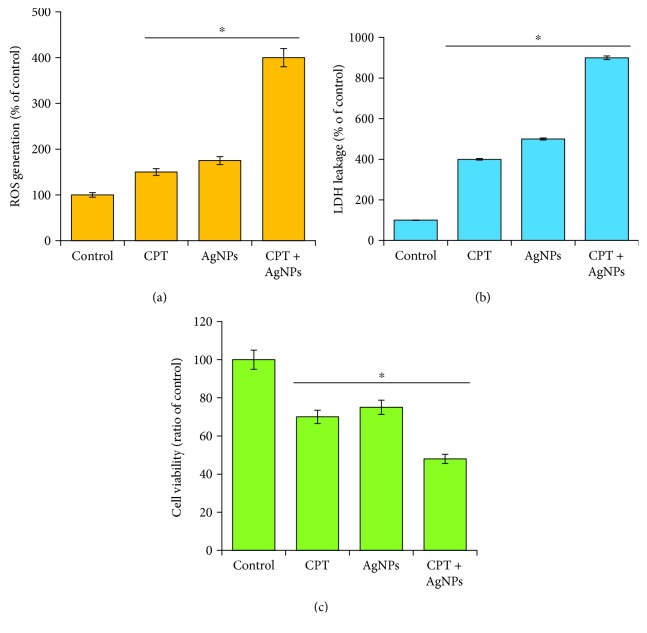
Combination effect of CPT and AgNPs on cytotoxicity in HeLa cells. HeLa cells were incubated with CPT (1 *μ*M), AgNPs (1 *μ*M), or a combination of CPT (1 *μ*M) and AgNPs (1 *μ*M) for 24 h. (a) The levels of ROS were assessed by measuring the relative fluorescence of 2′,7′-dichlorofluorescein using a spectrofluorometer. (b) The activity of LDH was measured at 490 nm using the LDH cytotoxicity kit. (c) The level of the dead-cell protease was determined by CytoTox-Glo cytotoxicity assay. The results are expressed as the mean ± standard deviation of three independent experiments. Differences between the treated and control groups were measured using Student's *t*-test. Statistically significant differences between the treated and control group are indicated by (^∗^*p* < 0.05).

To explore the relative contribution of each agent to the synergism, the effect of CPT and AgNPs inducing cytotoxicity was evaluated by equal dose of CPT (1 *μ*M) or AgNPs (1 *μ*M) and a combination of both CPT and AgNPs (1 *μ*M plus 1 *μ*M) and tested by measuring various cellular assays such as ROS generation, MDA level, LDH leakage, and dead-cell protease activity. Previous study suggested that the lethal effect of topoisomerase-I (topo-I) inhibitor CPT and topotecan on the cancer cells could be related to oxidative stress [57] and AgNPs-induced cell death due to enormous production of ROS and ultimately caused an imbalance of redox homeostasis in the cell [58, 59]. ROS are responsible for maintaining redox homeostasis in cells. Oxidative stresses are orchestrated by the level of pro- and antioxidant levels in the cellular system. Experiments were therefore performed to determine whether a similar mechanism might underlie the synergistic cytotoxic effects mediated by CPT and AgNPs. As shown in Figure 7(a), CPT and AgNPs combination induced a significant increase in ROS levels, compared with single-agent treatments, and relative to control untreated cells. Our findings suggested that ROS generation strikingly increased in cells when the cells were simultaneously exposed to CPT and AgNPs. While H209 and H526 SCLC cell lines treated with vorinostat and topotecan showed an increased level of phosphorylated form of H2AX (*γ*H2AX), enhanced level of ROS, and destruction of mitochondrial membrane suggesting a potential mechanism synergistic antitumor interaction between two anticancer compounds [57]. CPT- and AgNPs-induced ROS increased the cytotoxic activity and cellular changes in cancer cells *via* inducing apoptosis [60, 61].

Measurement of leakage of LDH, which is a cytoplasmic enzyme, is a critical factor for cell survival. We measured the LDH to determine the combination effect of CPT and AgNPs. While cells were treated with cytotoxic agents, viable cells used to have intact plasma membranes, whereas death cells membrane was disrupted and all the cellular contents were leaked into the media. Cytotoxicity induced by CPT or AgNPs or combination of CPT and AgNPs was assessed by LDH leakage into the culture medium. Cells were treated with CPT for 24 h resulted in a significant increase in LDH release relative to the untreated cells. The level of LDH leakage was significantly higher compared to control or single treatment (Figure 7(b)).

Among testing several alternative assays for cytotoxicity, measuring the levels of ATP is the most sensitive, reliable, and convenient method for monitoring active cell metabolism. Particularly, measuring protease activities provides a useful tool to help uncover the mechanism of cell death. To determine the combination effect of CPT and AgNPs, HeLa cells were treated with CPT (1 *μ*M), AgNPs (1 *μ*M), or both CPT and AgNPs (1 *μ*M plus 1 *μ*M), and then we measured cell viability by measuring dead-cell protease activity. Interestingly, the cytotoxicity performance induced by the CPT or AgNPs or a combination of CPT and AgNPs on cell viability, ROS generation, and LDH assay corroborate with dead-cell protease activity. The viability ratio was significantly reduced in the presence of both CPT and AgNPs compared to single treatment (Figure 7(c)). All the cytotoxicity obtained from this study was in accordance with previously reported in various type of anticancer drug such as platinum nanoparticles and trichostatin A, salinomycin and AgNPs, gemcitabine and AgNPs, and cisplatin and graphene oxide silver nanoparticles nanocomposite against cancer cells [19, 23, 25, 27]. Collectively, all these findings indicate that oxidative stress-mediated cytotoxicity plays a significant functional role in the enhanced lethality induced by the CPT/AgNPs combination in HeLa cells.

### 3.8. Effect of CPT and AgNPs on Expression of Oxidative and Antioxidative Stress Markers in HeLa Cells

**Figure 8:**
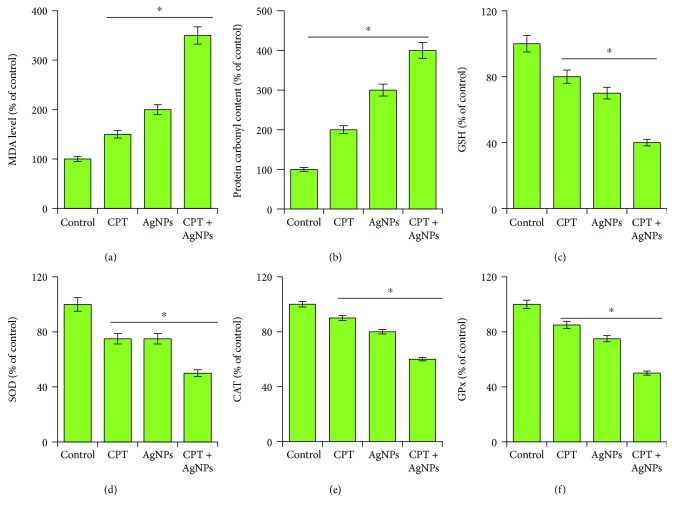
Combination effect of CPT and AgNPs on pro- and antioxidant markers in HeLa cells. HeLa cells were incubated with CPT (1 *μ*M), AgNPs (1 *μ*M), or a combination of CPT (1 *μ*M) and AgNPs (1 *μ*M) for 24 h. (a) The concentration of MDA was measured and expressed as nanomoles per milligram of protein. (b) The concentration of carbonyl content was measured and expressed as nanomoles per milligram of protein. (c) GSH concentration is expressed as mg/g of protein. (d) Specific activity of SOD is expressed as units per milligram of protein. (e) Specific activity of CAT is expressed as units per milligram of protein. (f) The specific activity of glutathione peroxidase (GPx) was expressed as unit per milligram of protein. Results are expressed as mean ± standard deviation of three independent experiments. There was a significant difference in treated cells compared to untreated cells with Student's *t*-test (^∗^*p* < 0.05).

In our study, we further extended our investigations to the effects of CPT and AgNPs on oxidative stress markers and antioxidants levels in HeLa cells. For oxidative stress markers, we measured malondialdehyde as a marker for lipid peroxidation and protein carbonyl content as an indicator of oxidative protein damage, which is the suitable marker for intracellular oxidative stress-dependent cellular damage [62]. Generally, high level of ROS leads to protein oxidation which converts proteins to protein carbonyl derivatives [63]. Oxidative lipid and protein damage cannot be repaired and causes irreversible modifications in lipids and proteins [64]. To measure the level of MDA and protein carbonyl content, the cells were treated with CPT (1 *μ*M), AgNPs (1 *μ*M), or both CPT and AgNPs (1 *μ*M plus 1 *μ*M) for 24 h. The level of MDA and carbonyl content dramatically increased in CPT- or AgNPs-treated cells, whereas the combination of CPT and AgNPs increased 4-fold compared to control cells (Figure 8). Similarly, while MCF-7 cell lines were treated with Adriamycin or topotecan, the lipid peroxidation level was significantly increased [62, 65]. It is well known that nanoparticles like palladium and AgNPs could increase the level of MDA in a variety of cancer cells including human cervical cancer cells and ovarian cancer cells [15, 23].

Since oxidative stress arises due to loss of balance between free radical or reactive oxygen species production, we are interested to measure various antioxidant systems in cells treated with CPT (1 *μ*M), AgNPs (1 *μ*M), or both CPT and AgNPs (1 *μ*M plus 1 *μ*M) for 24 h, such as glutathione (GSH), which is an important molecule for antioxidative defense and activities of antioxidant enzymes including superoxide dismutase (SOD), catalase (CAT), and glutathione peroxidase (GPx). Reduced glutathione (GSH) is the most abundant cellular thiol compound and an important intracellular antioxidant [66]. The level of GSH, SOD, CAT, and GPx were significantly decreased in CPT- or AgNPs-treated cells or a combination of CPT and AgNPs compared to untreated cells (Figure 8). GSH played a significant role in cell differentiation, proliferation, and apoptosis [67]. Similarly, reduced level of GSH was observed in topotecan-treated MCF-7 cells [62]. These data were consistent with the previous report suggesting that the increased oxidative stress incubated with a similar analog compound such as topotecan in various cancer cell lines including MCF-7 [62] and SMMC-7721 human hepatoma cell line with docetaxel [68]. The decreased level of all tested antioxidant markers such as GSH, SOD, CAT, and GPx might be due to increased oxidation of thiol groups to overcome the increased level of ROS in cells incubated with either CPT or AgNPs or a combination of CPT and AgNPs. SiHa cells were treated with 0.5 ∼ 5 *μ*M camptothecin (CPT) for 24 h; the cellular GSH content was decreased in a dose-dependent manner up to 52% [69].

At the early phase of apoptosis, ROS level was maintained by GSH; once the level of ROS leads to an alarming rate, it leads to an imbalance of redox homeostasis in the cells. Previously, several studies demonstrated that the combination of various anticancer drug like gemcitabine, cisplatin, and HDAC inhibitors such as trichostatin A and tubastatin A with AgNPs potentially increased the level of oxidative stress markers and decreased antioxidative stress markers in a variety of cancer cells including human cervical, breast, and ovarian cancer cells [23, 25, 27]. These findings suggest that the combination of CPT and AgNPs increases the oxidative stress in HeLa cells, which is a key mechanism of cytotoxicity.

### 3.9. Combination of CPT and AgNPs Induce Loss of Mitochondrial Function and Activation of Caspases

**Figure 9:**
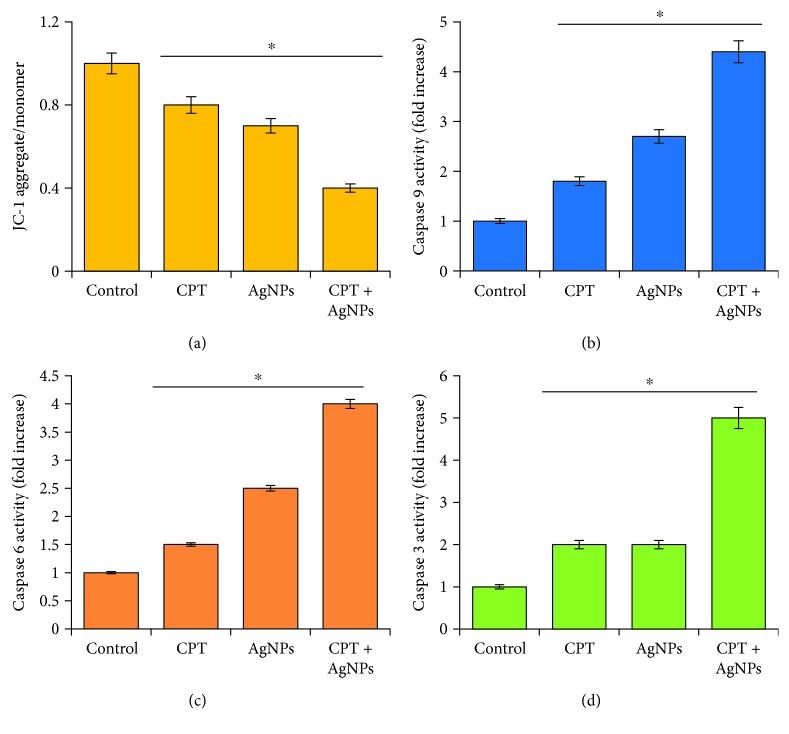
The effect of CPT, AgNPs, or a combination of CPT and AgNPs on MMP and caspase 9, 6, and 3. HeLa cells were incubated with CPT (1 *μ*M), AgNPs (1 *μ*M), or a combination of CPT (1 *μ*M) and AgNPs (1 *μ*M) for 24 h. (a) MMP (measured as a ratio of JC-1 aggregate to monomer) was determined after the treatments. (b–d) HeLa cells were incubated with CPT (1 *μ*M), AgNPs (1 *μ*M), or a combination of CPT (1 *μ*M) and AgNPs (1 *μ*M) for 24 h with and without a specific inhibitor of caspase. The concentration of P-nitroaniline released from the substrate was calculated from the absorbance at 405 nm. Results are expressed as mean ± standard deviation of three independent experiments. There was a significant difference in treated cells compared to untreated cells with Student's *t*-test (^∗^*p* < 0.05).

Imbalance level of pro- and antioxidant level could cause apoptosis. Generally, apoptosis takes place by two different pathways such as extrinsic and intrinsic pathways. The intrinsic pathway is mainly regulated by mitochondria, which is not only the site where antiapoptotic and proapoptotic proteins interact and determine cell fates but also the origin of signals that initiate the activation of caspases through various mechanisms. Furthermore, the mitochondrion is an important cellular source for the generation of reactive oxygen species (ROS) inside cells, which serves as inducing signals for apoptosis [70]. The optimum level of mitochondrial membrane potential (△*ψ*m) is an important factor for cell survival; the loss of mitochondrial membrane potential is a characteristic feature of the early phase of apoptosis. Previous study suggests that the reduction of cellular GSH levels increases the sensitivity of neurons to toxic insults and induces changes in mitochondrial function [71]. To verify that the reduced level of GSH and other antioxidant proteins level could alter mitochondrial membrane potential in HeLa cells, based on this conceptual idea, we examined the level of MMP in HeLa cells treated with CPT (1 *μ*M), AgNPs (1 *μ*M), or both CPT and AgNPs (1 *μ*M plus 1 *μ*M) for 24 h by using JC-1 probe. The results from this study depicted that the combination of CPT and AgNPs causes a dramatic loss of MMP compared to single treatment and untreated cells (Figure 9(a)). For instance, Jurkat cells treated with CPT resulted in a transient mitochondrial hyperpolarization and low MMP and underwent apoptotic cell death [72]. CPT inhibits cellular respiration; as a result, the MMP level was altered in *Leishmania donovani* [73]. AgNPs caused a remarkable loss of MMP in various types of cancer cells as well as a number of mitochondria including human lung cancer cells, neuroblastoma, and ovarian cancer cells [27, 53]. Our study was in accordance with the previously published report and suggested that increased level of ROS could alter the levels of intracellular antioxidants, such as GSH, NADH, or NADPH, which impair mitochondrial function [74]. The oxidation and depletion of cellular GSH can modulate the opening of the mitochondrial permeability transition pore [71, 74]. Therefore, the loss of antioxidants in the mitochondria could trigger the apoptotic pathway in the presence of CPT or AgNPs or a combination of CPT and AgNPs [71]. The results from this experiment along with the loss of cell viability, the increased level of ROS, and the decreased level of antioxidants in cells treated with CPT and AgNPs were eventually responsible for apoptosis.

Intrinsic-mediated apoptosis is mainly regulated by mitochondria, which results in apoptosome formation, activation of caspase-9, and subsequent activation of effector caspases [75]. The alteration of MMP is responsible for the release of Ca^2+^and cytochrome *c* and activation of caspases, which results in cell death [76–78]. CPT analogs exhibited toxic effects against cancer cells by inducing the activation of apoptotic caspases and formation of ROS [42–44]. Caspases activation is a crucial mechanism for apoptosis, and caspases are considered to be the “executioners” of cell death [79]. In order to determine the combinatorial effect of CPT and AgNPs on activation of caspases such as 9, 6, and 3, HeLa cells were treated with CPT (1 *μ*M), AgNPs (1 *μ*M), or both CPT and AgNPs (1 *μ*M plus 1 *μ*M) for 24 h and then measured the activity of caspase. The caspase activation was remarkably increased in the presence of CPT or AgNPs or a combination of both CPT and AgNPs (Figures 9(b)–9(d)). The results from this experiment were consistent with the earlier report and suggested that the combination of anticancer drug like gemcitabine, cisplatin, and HDAC inhibitors like trichostatin A and tubostatin A with AgNPs could increase activation of caspase 3 in a variety of cancer cells [23, 26, 27, 29]. Similarly, a combination of resveratrol and genistein induced apoptosis by enhancing the activities of caspase-9 and caspase-3 in HeLa cells via lowered mitochondrial membrane potential [80]. The results from this study suggest that the combination of CPT and AgNPs did have a synergistic effect on caspase-9, -6, and -3 activation in HeLa cells. The combined effect of CPT and AgNPs on activation of caspases was higher than that of single treatment.

### 3.10. CPT and AgNPs Upregulate Proapoptotic and Downregulate Antiapoptotic Gene Expressions

**Figure 10:**
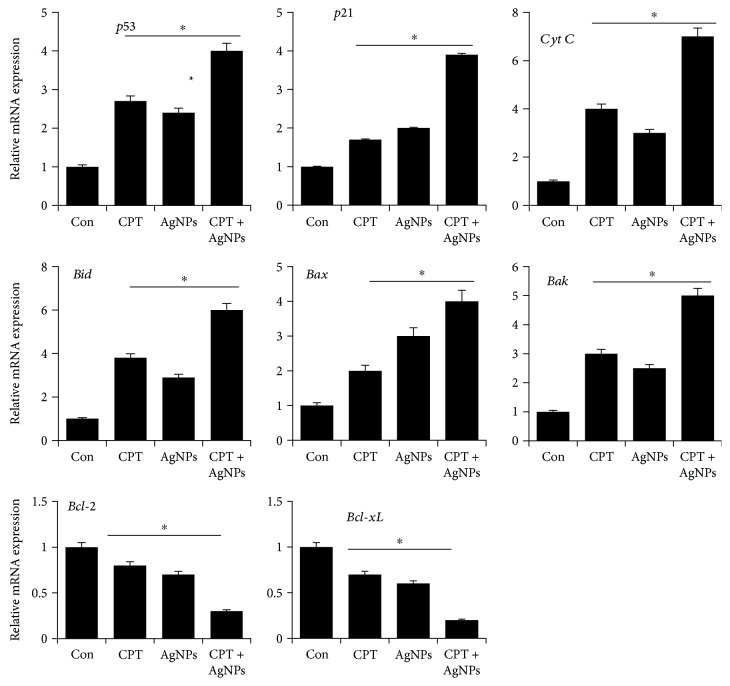
Effect of combined CPT and AgNPs treatment on the expression of proapoptotic and antiapoptotic genes expression in HeLa cells. HeLa cells were incubated with CPT (1 *μ*M), AgNPs (1 *μ*M), or a combination of CPT (1 *μ*M) and AgNPs (1 *μ*M) for 24 h, and then the relative expression levels of proapoptotic and antiapoptotic genes were analyzed by qRT-PCR. The results are expressed as mean ± standard deviation of three separate experiments. The treatment groups showed statistically significant differences from the control group, as determined by Student's *t*-test (^∗^*p* < 0.05).

Most chemotherapeutic drugs induce tumor cell death by apoptosis involving activation of caspases, and Bcl-2 family of proteins including anti- and proapoptotic molecules, and regulate cell sensitivity mainly at the mitochondrial level, and BCL-2 protein family members are key players in the response of tumor cells to a broad range of commonly used anticancer therapeutics [81, 82]. p53 is activated in a specific manner by posttranslational modifications and leads to DNA repair, cell cycle arrest, apoptosis, or cellular senescence based on the signals induced by DNA damage, hypoxia, or activated oncogenes [83]. Previous studies showed that cancer chemotherapeutic drugs, including doxorubicin, camptothecin, and cisplatin, are able to arrest tumor cells by initiating premature senescence *in vitro* and *in vivo* [84]. To determine the involvement of various pro- and antiapoptotic genes in CPT and AgNPs induced apoptosis, HeLa cells were treated with CPT (1 *μ*M), AgNPs (1 *μ*M), or both CPT and AgNPs (1 *μ*M plus 1 *μ*M) for 24 h. The expression level of proapoptotic genes such as p53, p21, Cyt C, Bid, Bax, and Bak was increased whereas Bcl-2 and Bcl-xL expression were decreased. The combination of CPT and AgNPs significantly increased all tested proapoptotic genes by 4-5-fold over the control. Conversely, a combination of CPT and AgNPs significantly downregulated antiapoptotic genes expression by 4-fold over the control (Figure 10). First, we examined p53 expression in the presence of CPT and AgNPs, which is the most potent and sequence-specific transcription factor, which induces cell cycle arrest, apoptosis, or senescence [85]. p21 is a transcriptional target of p53 and can act as a cell cycle inhibitor, it functions independently in response to a variety of stresses, including DNA damage [86]. Interestingly, p21 could regulate p53 function either negatively or positively for the induction of apoptosis [87]. CPT caused a sustained increase in the p21CIP1 protein levels in human breast cancer cells [88]. CPT induced apoptosis in Jurkat cells by increasing mitochondrial cytochrome c levels and mitochondrial hyperpolarization [72].

Our studies also gained the evidence that treating the cells with CPT or AgNPs or a combination of CPT and AgNPs increased expression of p53, p21, Cyt C, Bid, Bax, and Bak and downregulated expression of Bcl-2 and Bcl-xL. The combination of salinomycin and AgNPs upregulated the expression of p53, p21, Bax, Bak, caspase 9, and caspase 3 and downregulated the expression of Bcl-2 [15]. Furthermore, Gurunathan et al. [58] reported that biologically synthesized AgNPs induced apoptosis through upregulation of p53, Erk1/2, and c-procaspase-3 and downregulation of Bcl-2 proteins in human breast cancer cells. Collectively, these results indicate that both CPT and AgNPs synergistically working together in the regulation of p53 mediated apoptotic pathway in HeLa cells.

### 3.11. CPT and AgNPs Modulate Various Signaling Molecules Involved in Cell Survival Cytotoxicity and Apoptosis

**Figure 11:**
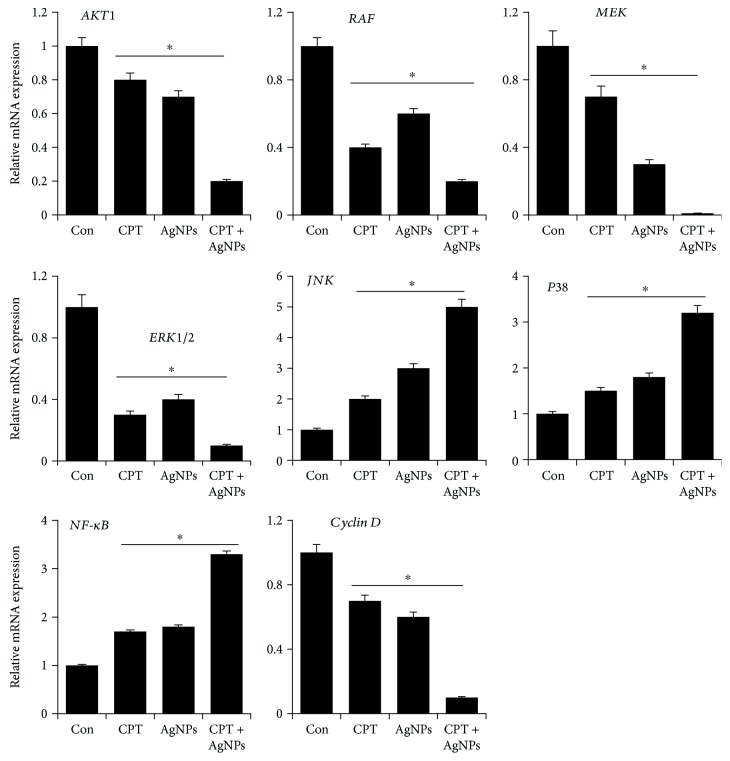
Reverse transcription-quantitative polymerase chain reaction (RT-qPCR) analysis of expression of various genes involved in signaling pathways of cell survival, cytotoxicity, and apoptosis. The expression pattern of various genes involved in signaling pathways was analyzed by exposure of HeLa cells to CPT (1 *μ*M), AgNPs (1 *μ*M), or a combination of CPT (1 *μ*M) and AgNPs (1 *μ*M) for 24 h. After 24 h treatment, expression fold level was determined as fold changes in reference to expression values against GAPDH. Results are expressed as fold changes. At least three independent experiments were performed for each sample. The treated groups showed statistically significant differences from the control group by the Student's *t*-test (^∗^*p* < 0.05).

In order to understand the functional role of various signaling molecules involved in cell survival, cytotoxicity and apoptosis in the presence of CPT and AgNPs were analyzed. Therefore, we measured the expression level of Akt1, RAF, MEK, Erk1/2, JNK, P38, NF-*κ*B, and Cyclin D in HeLa cells by treating cells with CPT (1 *μ*M), AgNPs (1 *μ*M), or both CPT and AgNPs (1 *μ*M plus 1 *μ*M) for 24 h. PI3K/Akt signals play an important role in various cellular processes such as cell growth, survival, cell size, response to nutrients, proliferation, migration, invasion, angiogenesis, and tumor progression [89]. Akt has direct effects on the apoptosis machinery. The results from our findings clearly indicated that CPT or AgNPs or a combination of both CPT and AgNPs inhibited expression of Akt by fold compared to control (Figure 11). For instance, a conjugate of CPT and a somatostatin analog inhibits cell invasion by inhibition of phosphorylation of Akt against prostate cancer via a possible involvement of PI3K/Akt signaling pathway [90]. Similarly, AgNPs inhibits phosphorylation of Akt in the VEGF-induced angiogenic process such as cell survival and migration in bovine retinal endothelial cells [51, 52]. Collectively, the data from Akt expression suggest that combination of CPT and AgNPs significantly reduce the cell survival than single treatment.

Raf kinase involved in the regulation of Ras/Raf/MEK/ERK kinase cascade, which is a critical player to transfers mitogenic signals from the cell membrane to the nucleus. All these kinases are particularly involved in epidermal growth factor receptor (EGFR) family involved in certain cancer and are constitutively active in certain cancers, and this promotes uncontrolled cell growth [91]. RAF can activate phosphorylation of MEK1 and MEK2. MEK1 and MEK2, in turn, activate the ERK1 and ERK2 mitogen-activated protein (MAP) kinases via phosphorylation. Thus, RAF proteins are crucial regulators of the ERK MAP kinase signaling cascade involving in cell proliferation, differentiation, migration, and survival [92]. Based on the conception derived from literature, we examined the expression of RAF, MEK, and ERK1/2 in CPT (1 *μ*M), AgNPs (1 *μ*M), or both CPT and AgNPs (1 *μ*M plus 1 *μ*M) exposed HeLa cells for 24 h. The results clearly indicated that all these Raf/MEK/ERK kinase cascade molecules are significantly downregulated between 1 and 4-fold level either in a single treatment or in a combination treatment (Figure 11). These findings suggest that CPT and AgNPs work closely to reduce the cell survival and increase the apoptosis by regulating the Raf/MEK/ERK kinase cascade.

In order to reveal the role of the mitogen-activated protein kinases including JNK and p38 in CPT and AgNPs induced cytotoxicity and apoptosis, next we examined the impact of CPT and AgNPs on JNK and p38 kinase. We performed the expression analysis of JNK and p38 in HeLa cells after exposed to CPT (1 *μ*M), AgNPs (1 *μ*M), or both CPT and AgNPs (1 *μ*M plus 1 *μ*M) for 24 h. CPT and AgNPs exhibited the upregulation of JNK and p38 by 1–5-fold, which accelerated and enhanced apoptosis (Figure 11). According to previous studies, both JNK and p38 MAPK are key mediators of stress signals and inflammatory responses evoked by a variety of agents such as UV- and gamma-irradiation, heat shock, osmotic stress, and inflammatory cytokines. Further studies suggested that the strong activation of JNK and p38 MAPK in cells treated with several stress signals ultimately lead to apoptosis [93–95]. Consistent with previous findings, AgNPs activated stress signals and induced apoptosis in a variety of cancer cells including human breast cancer cells and F9 teratocarcinoma stem cells [58, 96]. NF-*κ*B seems to be an important gene playing an important role in anti- and proapoptotic functions and was found to be one of the main determinants of the cellular outcome after treatment with nanoparticles [97, 98]. When HepG2 cells were exposed to AgNPs, a higher level of NF-*κ*B activity and strong upregulation of the mRNA level of some of the NF-*κ*B-related genes were found, whereas there are no significant changes in A549 cells [99]. CPT activates NF-*κ*B by a mechanism that is dependent on initial nuclear DNA damage followed by cytoplasmic signaling events [100]. Piret and Piette [101] demonstrated that DNA damaging agents increases NF-*κ*B activation, which correlates with their capacity to induce DNA breaks. Samuel et al. [102] observed that CPT could induce the expression of NF-*κ*B in SW480 colon cancer cells in a dose-dependent manner, but not in HCT116 cells. Interestingly, when SW480 colon cancer cells were exposed to CPT, NF-*κ*B was activated accompanied by secretion of the cytokine CXCL8, but not by upregulation of the antiapoptotic genes, cIAP2, or Bcl-XL. The data from our findings also correlates with earlier findings that CPT or AgNPs or a combination of CPT and AgNPs increases expression of NF-*κ*B along with decreasing expression of antiapoptotic proteins such as Bcl2 and Bcl-xL.

Cyclin D1 is a vital regulator of cell cycle progression and protooncogenes and regulates G1 to S phase progression in many different cell types and also functions as a transcriptional modulator by regulating the activity of several transcription factors. In mammalian cells, DNA damage-causing agents, environmental stress, and viral infection seem to induce the ubiquitin-dependent degradation of cyclin D1 [103]. To determine whether the combination of CPT and AgNPs could affect cyclin D1 gene expression levels and involved in apoptosis of HeLa cells, cells were treated with CPT (1 *μ*M), AgNPs (1 *μ*M), or both CPT and AgNPs (1 *μ*M plus 1 *μ*M) for 24 h. As a result, CPT or AgNPs or a combination of CPT and AgNPs downregulated the expression of cyclin D1 in HeLa cells.

Previous study suggests that therapeutic agents have been observed to induce cyclin D1 degradation in human breast carcinoma cell lines [104]. Cyclin D1 proteasomal degradation is the possible anticancer. The data from this study suggest that the combination of CPT and AgNPs is able to inhibit the cell viability which is associated with apoptosis, and cell cycle arrest correlated with reduced amounts of antiapoptotic genes Bcl-2, Bcl-xL, and cyclin D1 and increased expression of JNK, P38, and NF-*κ*B. Taken together, our results clearly suggest that the downregulation of PI3K/Akt and Raf/MEK/ERK1/2 and upregulation of JNK and p38 MAPK signaling pathways are crucial in the context of DNA-damaging drug-induced apoptosis as well as AgNPs induced apoptosis, and this concluded that the activation of JNK/P38 pathway may be involved in the apoptosis induced synergistically by topoisomerase I inhibitor and AgNPs via mechanism of oxidative stress.

## 4. Conclusion

Nanoparticles mediated cancer therapy plays an important role to increase therapeutic efficiency of cancer by a combination of nanoparticles and anticancer drug in tumor control and reduce undesired side effects by improving the pharmacokinetics and tumor deposition of the drug payloads. AgNPs are known to exhibit anticancer activity and DNA-topoisomerase I inhibitor such as CPT analogs exhibit toxic effects against cancer cells by inducing the activation of apoptotic caspases and formation of ROS and ultimately increases anticancer activity. To explore the possibility of the combination effect of CPT and AgNPs in HeLa cells, initially, the cells were treated with various concentrations of CPT and AgNPs; the results depicted that both CPT and AgNPs induce cell death dose-dependently. The combination of CPT and AgNPs significantly inhibits cell proliferation and increases cytotoxicity and apoptosis by increasing ROS generation and leakage of LDH and also altering mitochondrial membrane potential and activation of caspase 9, 6, and 3. Further, it increases the level of prooxidants such as MDA and protein carbonyl content and decreases the level of antioxidants such as GSH, SOD, CAT, and GPx. Interestingly, the combined treatment increases the expression of proapoptotic genes including p53, p21, Cyt C, Bid, Bax, Bak and antiapoptotic genes such as Bcl-2 and Bcl-xL. Finally, the combination of CPT and AgNPs modulates the expression of various signaling molecules involved in cell survival, cell viability, cell proliferation, cytotoxicity, and apoptosis such as Akt1, RAF, MEK, Erk1/2, JNK, P38, NF-*κ*B, and Cyclin D. The findings from this study suggest that combination of CPT and AgNPs at lower doses could potentially induce cytotoxicity and apoptosis without any undesired cytotoxic effects, which can increase the efficacy of two different molecules for cancer treatment. Therefore, the combination of nanoparticles and anticancer drug presents as a promising approach for cancer research. In practice, combination chemotherapy results in a better response and improved survival compared with single-agent therapy.

## Figures and Tables

**Table 1:** Primer list for real-time polymerase chain reaction.

Gene	Primer
*Bax*	F: GAG AGG TCT TTT TCC GAG TGG
	R: GGA GGA AGT CCA ATG TCC AG

*p53*	F: AGG AAA TTT GCG TGT GGA GTA T
	R: TCC GTC CCA GTA GAT TAC CAC T

*Bak*	F: CTC AGA GTT CCA GAC CAT GTT G
	R: CAT GCT GGT AGA CGT GTA GGG

*Bcl-2*	F: CTG AGT ACC TGA ACC GGC A
	R: GAG AAA TCA AAC AGA GGC CG

*p21*	F: ATG TGG ACC TGT CAC TGT CTT G
	R: CTT CCT CTT GGA GAA GAT CAG C

*Cyt C*	F: GCGTGTCCTTGGACTTAGAG
	R: GGCGGCTGTGTAAGAGTATC

*Bid*	F: CCTTGCTCCGTGATGTCTTTC
	R: GTAGGTGCGTAGGTTCTGGT

*Bcl-xL*	F: GTAAACTGGGGTCGCATTGT
	R: CGATCCGACTCACCAATACC

*GAPDH*	F: AACGGATTTGGTCGTATTGGG
	R: TCGCTCCTGGAAGATGGTGAT

**Table 2:** Primer list for real-time polymerase chain reaction.

Gene	Primer
*AKT1*	F: AGGTGACACTATAGAATAGAGGAGATGGACTTCCGGTC
	R: GTACGACTCACTATAGGGAAGGATCTTCATGGCGTAGTAGC

*ERK1/2*	F: AGGTGACACTATAGAATAGGAGCAGTATTACGACCCGA
	R: GTACGACTCACTATAGGGAGATGTCTGAGCACGTCCAGT

*JNK*	F: AGGTGACACTATAGAATACAGAAGCTCCACCACCAAAGAT
	R: GTACGACTCACTATAGGGAGCCATTGATCACTGCTGCAC

*p38*	F: AGGTGACACTATAGAATATTCAGTCTTTGACTCAGATGCC
	R: GTACGACTCACTATAGGGAGTCAGGCTTTTCCACTCATCT

*RAF1*	F: AGACTGCTCACAGGGCCTTA
	R: CTGCAAATGGCTTCCTTCTC

*MEK*	F: GGTGTTTATTGGGCCTCAGA
	R: ACCCGGAGCATCACAAATAG

*Cyclin D*	F: ACAAACAGATCATCCGCAAACAC
	R: TGTTGGGGCTCCTCAGGTTC

*NF-κB*	F: AGGTGACACTATAGAATAGCGGGCGTCTAAAATTCTG
	R: GTACGACTCACTATAGGGATTCCACGATCACCAGGTAGG

## Data Availability

The data used to support the findings of this study are available from the corresponding author upon request.
